# Relationship between smartphone addiction and eating disorders and lifestyle among Chinese college students

**DOI:** 10.3389/fpubh.2023.1111477

**Published:** 2023-05-19

**Authors:** Jun Wang, Qing-Hong Hao, Wei Peng, Yang Tu, Lan Zhang, Tian-Min Zhu

**Affiliations:** ^1^Chengdu Women's and Children's Central Hospital, School of Medicine, University of Electronic Science and Technology of China, Chengdu, China; ^2^Longgang District Maternity and Child Healthcare Hospital of Shenzhen City, Shenzhen, China; ^3^Chongqing Hospital of Traditional Chinese Medicine, Chongqing, China; ^4^Chongqing University Cancer Hospital, Oncology Treatment Center of Traditional Chinese Medicine, Chongqing, China; ^5^School of Rehabilitation and Health Preservation, Chengdu University of Traditional Chinese Medicine, Chengdu, China

**Keywords:** smartphone addiction, eating disorders, relationship, college students, lifestyle

## Abstract

**Purpose:**

Smartphone addiction has been a matter of serious concern among society and parents because of its high incidence and serious negative effects. This study aimed to determine the association between smartphone addiction and eating disorders and lifestyle changes among college students.

**Methods:**

The present article is a descriptive, cross-sectional study involving 1,112 college students from several universities in Chengdu, China. The data were collected by using the Chinese version of the Mobile Phone Addiction Index (MPAI) and the Eating Attitudes Test-26 (EAT-26). In addition, the information on sociodemographic, lifestyle, and smartphone use were obtained through a self-administered questionnaire.

**Results:**

The prevalence of smartphone addiction among the students involved in the study was 22.6%, of which 10.4% were at risk for eating disorders. Female students had higher MPAI scores and EAT-26 scores than male students (*p* < 0.001). The proportion of male students with a risk of eating disorders was significantly higher than that of female students (*p* < 0.05). The total EAT-26 scores of students with smartphone addiction were higher than that of others (*p* < 0.001). The correlation analysis indicated that the MPAI scores were significantly positively correlated with the EAT-26 scores, depression and anxiety, difficulty in falling asleep at night, the frequency of eating fast food and drinking carbonated soft drink (*p* < 0.01). In addition, the MPAI scores were significantly negatively correlated with skipping breakfast and the frequency of physical activity (*p* < 0.05).

**Conclusion:**

Smartphone addiction is significantly associated with eating disorders, eating habits, and lifestyle. The influence of dietary habits and lifestyle needs to be considered for the prevention and development of an intervention for smartphone addiction among college students.

## Introduction

The Internet has become an increasingly important aspect of human life. It is an essential contemporary way of transmitting information and making connections with peers, friends, and family, especially among adolescents (1–3). According to the report, as of 2021, there were 4.66 billion Internet users worldwide, and the number of Chinese Internet users was as high as 989 million (approximately 71% of the country's total population) (4). Smartphones are one of the most convenient and easiest ways to surf the Internet. As a relatively free group, college students who have a poor ability to resist temptation are more prone to overdependence on smartphones. Smartphone addiction seriously affects college students' academic performance, energy level, and eating and exercise habits and brings on more anxiety, depression, insomnia, emotional imbalance, and even suicide due to cyberbullying and Internet fraud (5, 6).

Previous studies have focused on the relationship between smartphone addiction and depression, anxiety, study stress, insomnia, physical symptoms (musculoskeletal pain), and health risk behaviors such as smoking, drinking, and suicide (7–13). A systematic review suggested that smartphone addiction is closely associated with mental health, and depression and anxiety are mediators of mental health problems (11). Furthermore, research has documented that individuals with smartphone addiction are more likely to postpone their bedtime, suffer from insomnia, have depression and anxiety (7, 8, 10, 12), and have a higher likelihood of suicidal ideation and suicide attempts (14). Some scholars have indicated that smartphone addiction can cause pain in certain extremities (12, 13). In addition, the researchers found that study, stress loneliness, and interpersonal problems are important factors for smartphone addiction (9, 15). In recent years, there has long been an interest in the relationship between behavior and diet. The relationship between eating behavior and smartphone addiction has become a heated topic under investigation, and there are only a few relevant reports in China; therefore, it is necessary to continue to carry out relevant research.

Eating disorders are serious mental disorders characterized by persistent disturbed eating behaviors, which mainly manifested as excessive preoccupation with diet and body shape (16, 17). Eating disorders are closely related to dietary attitude and, social, cultural, and psychological circumstances (18). Lifestyle factors (i.e., physically active, sleep duration, dietary behavior) can make a major difference in physical and mental health. Unhealthy lifestyles can increase the risk of cardiovascular, nervous, and endocrine diseases (19, 20). University students are more likely to become confused, lonely, helpless, unconfident, and burdened by different expectations and demands (21, 22). As a result, they are more inclined to develop problematic behaviors, such as unhealthy eating, smoking, and drinking (23, 24).

Furthermore, a healthy lifestyle and eating habits are closely associated with physical and mental health. Several studies found that Internet addiction (IA) has adverse effects on eating disorders and lifestyle characteristics among adolescents (24–28). One previous study showed that the Mobile Phone Addiction Index (MPAI) was positively associated with sugar-sweetened food intake (29). Kim et al. (26) in a study involving 853 Korean junior high school students confirmed that the proportion of irregular bedtimes was higher among high-risk Internet users than non-risk Internet users, and high-risk Internet users had more irregular dietary behavior, a high frequency of skipping meals, and snacking. A number of studies suggested that IA is closely associated with sleep disturbance (30–32). HR et al. (33) in a sample of 928 students from southwestern Iran confirmed that IA directly affects BMI and fast food consumption. A recent study based on Bangladesh adults showed a higher prevalence of IA among physically inactive participants (34). A nationwide cross-sectional school-based health survey based in Malaysia showed a significant correlation between the frequency of drinking carbonated soft drinks, the frequency of eating fast food, and sedentary behavior and IA (35).

Smartphones are the most popular tool for Internet activities. However, reports of the relationship between smartphone addiction and eating disorders as well as lifestyle are still lacking in China. Therefore, the present study aimed to further elucidate the link between smartphone addiction and eating disorders and lifestyle in college students.

## Methods

### Study design and participants

This study was conducted using a cross-sectional design with cluster convenience sampling at several universities in Chengdu, Sichuan Province, China, from August 2021 to November 2021.

The formula for calculating the sample size for a population is as follows (36, 37):


$$n={\left(\frac{Z\alpha /2}{\delta }\right)}^{2}\pi (1-\pi )={\left(\frac{1.96}{0.03}\right)}^{2}0.3\times 0.7=\;896$$

In the formula, *n* denotes the size of the population, *Z*α denotes a 95% confidence interval, δ indicates the margin of error, and π denotes the incidence of the health risk behaviors of college students. Referring to the relevant domestic literature (38–41), in this study, Z = 1.96, α = 0.05, δ = 0.03, and π = 0.3. Taking into account the 10% dropout rate, the actual sample size was 1,133. From the sample size, 21 were excluded because of their refusal to participate or the lack of questionnaire data. The inclusion criteria of this study were as follows: (1) being a smartphone user, (2) age between 17 and 29 years, (3) being in good physical health, and (4) willing to sign a formal consent to participate. Students with substance abuse, who were pregnant or lactating, and any chronic or acute disease, such as diabetes, kidney disease, cardiovascular diseases, and cancer, were excluded from this study. All students were informed of their rights to participate, and they could withdraw at any time. The anonymous, self-reported questionnaires were distributed or delivered online through a well-known Chinese social media app (WeChat, Ten-cent Inc., Shenzhen, China) to college students.

### Ethics statement

This study protocol was approved by the Ethics Committee of the Affiliated Hospital of Chengdu University of Traditional Chinese Medicine (CDUTCM). Informed consent was obtained from all the participants.

### Measures

Our questionnaires consisted of basic characteristics, eating habits, lifestyle, health, and smartphone use.

#### Mobile phone addiction index

The Chinese version of MPAI (42, 43) contained 17 items with a five-point Likert scale (1 = “not at all”, 2 = “occasionally”, 3 = “frequently”, 4 = “often”, and 5 = “always”). If the participants responded to 8 or more questions with a rating of 4 or above, they were defined as having mobile phone addiction. The higher the score of MPAI, the greater the level of mobile phone dependence. The MPAI has been validated and has good internal consistency. The Cronbach's alpha was 0.870 (44).

#### The eating attitudes test-26

The EAT-26 scale (45) is a standardized self-report scale of anorexic/bulimic attitudes and beliefs with 26 items. It consists of three sections: diet, bulimia and food preoccupation, and oral control. The answer for each item uses a six-point Likert scale which is grouped into a 4-point format (0 = “never/rarely/sometimes”, 1 = “often”, 2 = “usually”, and 3 = “always”). The total score ranges from 0 to 78. Participants with scores of 20 or higher were considered to be at a high risk of eating disorders. The higher the EAT-26 score, the more the number of eating disorder symptoms. The Chinese version of EAT-26 has satisfied reliability and validity. The Cronbach's alpha was 0.843 (46).

#### Lifestyle variables

Referring to the relevant studies (35, 47, 48), in this study, the information on lifestyle included eating habits, physical activity, and sleep habits. Eating habits included taste preferences, the number of main meals per day, the frequency of eating fast food meals weekly, the frequency of eating late-night snacks monthly, and the frequency of drinking carbonated soft drinks monthly. Physical activity included the frequency of physical activity for a total of at least 60 min weekly and time spent sitting and doing other things per day, excluding sitting in school and for homework. Sleep habits included difficulty in falling asleep and sleep duration. The details are shown in the Supplementary material.

In addition, this study also investigated the emotional state (depression and anxiety) of college students through self-report. We also calculated BMI based on the height and weight that the college students filled in themselves. According to the Working Group on Obesity in China (49, 50), the BMI was divided into the following levels: underweight (< 18.5 kg/m^2^), normal weight (18.5–23.9 kg/m^2^), overweight (24–27.9 kg/m^2^), and obese (≥28 kg/m^2^).

### Statistical analysis

The data were analyzed using the IBM SPSS Statistics 23.0 software (Chicago, USA). The descriptive statistics were performed to report the analysis of the data that were displayed as mean and standard deviations (SD). The categorical data were expressed as frequencies or percentages. A two-sample *T*-test was used for data with normal distribution and the Mann–Whitney U test for the data without normal distribution. The chi-squared test was used for the categorical data. Pearson and Spearman correlation analyses were used to analyze the relationships between the examined variables and smartphone use. A multiple linear regression analysis was used to examine which variables were predictors of the MPAI scores. Statistical significance was set at a *p*-value of < 0.05.

## Results

### Demographics analyses

A total of 1,112 students were involved in this survey, with an overall response rate of 98.2%. The sociodemographic, lifestyle, and clinical characteristics of the students are shown in Table 1. The average age of the students was 21.4 ± 3.2. The average BMI of the students was 20.4 ± 2.5 kg/m^2^. A significant difference in the proportion of the normal BMI between male and female s was observed (*p* < 0.001). The proportion of students who were underweight was significantly higher in females than in males (12.2% in male students and 26.5% in female students, respectively) (*p* < 0.001); in addition, 10.6% of males and 5.0% of females were overweight and 1.8% of males and 0.4% of females were obese. The students who reported that they skipped breakfast were 40.4% and their frequency of main meals was 2.5 ± 0.6 times/day. There was no difference between males and females in the frequency of eating late-night snacks, which was 2.9 ± 0.7 times/day in males and 2.8 ± 0.7 times/day in females, respectively, while the frequency of eating fast food was higher in females (2.0 ± 1.1 times/week in females and 1.8 ± 1.1 times/week in males, respectively) (*p* < 0.001). Students with taste preferences accounted for 56%. The vast majority of students like spicy food (63.6%) and females prefer spicy food more than males (67.2% in females and 54.9% in males, respectively) (*p* < 0.01). The average number of times carbonated soft drinks were consumed by students was 1.3 ± 0.5 times/day.

**Table 1:** The sociodemographic, lifestyle and clinical characteristics of participants.

	**Male *N =* 433**	**Female *N =* 679**	**Total *N =* 1112**	***P***
Age (Mean ± SD)	21.5 ± 2.5	20.9 ± 3.6	21.4 ± 3.2	-
BMI (kg/m^2^) (Mean ± SD)	21.1 ± 2.6	20.0 ± 2.2	20.4 ± 2.5	< 0.001^***^
BMI (*n* %)				
Underweight	53(12.2)	180 (26.5)	233 (21.0)	< 0.001^***^
Normal weight	324 (74.8)	462 (68.0)	786 (70.7)	< 0.05^*^
Overweight	46 (10.6)	34 (5.0)	80 (7.2)	< 0.001^***^
Obese	8 (1.8)	3 (0.4)	11 (1.0)	< 0.05^*^
Depression and anxiety (*n* %)				0.068
**No**	293 (67.7)	423 (62.3)	716(64.4)	-
**Yes**	140 (32.3)	256 (37.7)	396 (35.6)	-
Slight	132 (94.3)	237 (92.6)	369 (93.2)	0.519
Moderate	8 (5.7)	18 (7.0)	26 (6.6)	0.613
severe	0	1 (0.4)	1 (0.2)	0.459
**Basic information of smartphone use**				
Smartphone age (year) (Mean ± SD)	5.6 ± 2.0	5.6 ± 2.0	5.6 ± 2.0	0.727
Time to use smartphone weekday (h/day) (Mean ± SD)	5.1 ± 2.0	5.6 ± 1.9	5.4 ± 1.9	< 0.001^***^
Time to use smartphone weekend (h/day) (Mean ± SD)	6.2 ± 2.0	6.7 ± 1.8	6.5 ± 1.9	< 0.001^***^
Time to stay on the internet (h/day) (Mean ± SD)	4.6 ± 2.1	4.7 ± 2.0	4.7 ± 2.0	0.344
Purpose of smartphone usage (*n* %)				
Music/Video/ Social networks	292 (67.4)	212 (31.2)	504 (45.3)	-
Game	270 (62.4)	198 (29.2)	468 (42.1)	-
shopping	185 (42.7)	502 (73.9)	687 (61.8)	-
Study	243 (56.1)	447 (65.8)	690 (62.1)	-
**Eating habits**				
Taste preferences (*n* %)				< 0.001^***^
**No**	249 (57.5)	240 (35.3)	489 (44.0)	-
**Yes**	184 (42.5)	439 (64.7)	623 (56.0)	-
Sour	25 (13.6)	43 (9.9)	68 (10.9)	0.166
sweet	38 (20.7)	75 (17.1)	113 (18.1)	0.292
spicy	101 (54.9)	295 (67.2)	396 (63.6)	< 0.01^**^
salty	20 (10.9)	26 (5.9)	46 (7.4)	< 0.05^*^
Skip breakfast (*n* %)				0.080
**No**	243 (54.9)	420 (61.9)	663 (58.7)	-
**Yes**	190 (42.9)	259 (38.1)	449 (40.4)	-
Frequency of main meals (times/day) (Mean ± SD)	2.5 ± 0.7	2.5 ± 0.7	2.5 ± 0.7	0.997
Frequency of fast-food (times/week) (Mean ± SD)	1.8 ± 1.1	2.0 ± 1.1	1.9 ± 1.1	< 0.001^***^
Frequency of late-night snack (times/month) (Mean ± SD)	2.9 ± 0.9	2.8 ± 0.7	2.9 ± 0.8	0.077
Frequency of carbonated soft drink (times/day) (Mean ± SD)	1.3 ± 0.5	1.3 ± 0.5	1.3 ± 0.5	0.736
**Physical activity and sleep habits**				
Regular physical activity(≥3day/week) (Mean ± SD)	156 (36.0)	119 (17.5)	275 (24.7)	< 0.001^***^
Sleep duration (h/day) (Mean ± SD)	7.1 ± 1.1	7.2 ± 1.1	7.2 ± 1.1	0.315
Difficulty in falling asleep at night (*n* %)				0.832
**Yes**	87 (20.1)	140 (20.6)	227 (20.4)	-
**No**	346 (79.9)	539 (79.4)	885 (79.6)	-

^*^p < 0.05, ^**^p < 0.01, ^***^p < 0.0001. Mann–Whitney U-test and chi-squared test analyses.

The average age for smartphone use was 5.6 ± 2.0 years. Although the time to stay on the Internet (h/day) was not different between males and females, the time to use a smartphone (h/day) of females was higher than that of males and the time to use a smartphone on weekends was significantly higher than that on weekdays (*p* < 0.001). The purpose of smartphone usage is to play games in males and shopping in females.

The proportion of students who believed they had depression and anxiety was 35.6 and 93.2% of them had mild and 6.6% of them had moderate depression and anxiety. In addition, 36.0% of males and 17.5% of females have been doing regular physical activity (≥ 3 days/week) (*p* < 0.001). Fewer students had difficulty in falling asleep, and there was no significant difference in the sleeping duration (h/day) between males and females.

### Test scores

Table 2 shows the total test scores and several related variables' analyses between males and females. The total scores of MPAI were significantly different between males and females (*p* < 0.001). The total scores of the EAT-26 scale were significantly higher in females than in males (7.4 ± 0.4 in male students and 8.7 ± 0.3 in female students, respectively) (*p* < 0.001).

**Table 2:** Assessment of eating disorders, mobile phone addiction test.

	**Male *N =* 433**	**Female *N =* 679**	**Total *N =* 1112**	***P***
Total score of MPAI (Mean ± SD)	43.8 ± 0.6	46.9 ± 0.5	45.7 ± 0.4	< 0.001^***^
Total score of EAT-26 (Mean ± SD)	7.4 ± 0.4	8.7 ± 0.3	8.2 ± 0.2	< 0.001^***^
**Smartphone addiction (Self report) (*****n*** **%)**				0.304
Yes	221 (51.0)	368 (54.2)	589 (53.0)	-
No	212 (49.0)	311 (45.8)	523 (47.0)	-
**Smartphone addiction (*****n*** **%)**				0.061
Yes	85 (19.6)	166 (24.5)	251 (22.6)	
No	348 (80.4)	513 (75.5)	861 (77.4)	
**Potential eating disorders (*****n*** **%)**				< 0.05^*^
Yes	56 (12.9)	60 (8.8)	116 (10.4)	-
No	377 (87.1)	619 (91.2)	996 (89.6)	-

MPAI, Mobile Phone Addiction Index; EAT-26, The Eating Attitudes Test-26. ^*^p < 0.05, ^**^p < 0.01, ^***^p < 0.0001. Independent sample t-test, Mann–Whitney U test and chi-squared test analyses.

More than half of the students consider themselves addicted to smartphone use (51% in male students and 54.2% in female students, respectively). It was found that 19.6% of males and 24.5% of females are addicted to smartphones, and nearly 10% of students believed that they have IA. The risk of eating disorders of males was significantly higher than that of females (12.9% in male students and 8.8% in females, respectively) (*p* < 0.05).

### Smartphone addiction

The students who self-reported that they were addicted to their smartphone accounted for 79.3% of the students with smartphone disorder. The proportion of students with smartphone disorder having depression and anxiety and difficulty in falling asleep (*p* < 0.001) was higher. The total EAT-26 scores of students with smartphone addiction were higher than those of others (*p* < 0.001). Students with smartphone addiction skipped breakfast significantly more often than normal subjects (*p* < 0.001). The frequency of drinking carbonated soft drinks was higher in the students with smartphone addiction than others (*p* < 0.001). Time to stay on the Internet, time to use a smartphone on weekends, and time to use a smartphone on weekdays (h/day) were significantly higher in the students with smartphone addiction (*p* < 0.001) (Table 3).

**Table 3:** Distribution of smartphone addiction and related variables.

	**Smartphone**	**Disorder**	
	**Yes** ***N** =* **251**	**No** ***N** =* **861**	***P***
Total score of EAT-26 (Mean ± SD)	10.0 ± 0.7	8.0 ± 0.2	< 0.001^***^
Depression and anxiety (*n* %)			< 0.001^***^
Yes	118 (47.0)	278 (32.3)	-
No	133 (53.0)	583 (67.7)	-
**Basic information of smartphone use**			
Smartphone addiction (Self report) (*n* %)			< 0.001^***^
Yes	199 (79.3)	390 (45.3)	-
No	52 (20.7)	471(54.7)	-
Time to use smartphone weekday (h/day) (Mean ± SD)	6.0 ± 0.2	5.3 ± 0.1	< 0.001^***^
Time to use smartphone weekend (h/day) (Mean ± SD)	7.4 ± 0.2	6.5 ± 0.1	< 0.001^***^
Time to stay on the internet (h/day) (Mean ± SD)	5.3 ± 0.2	4.6 ± 0.1	< 0.001^***^
**Eating habits**			
Taste preferences (*n* %)			0.857
**No**	83 (33.1)	406 (47.2)	-
**Yes**	168 (66.9)	455(52.8)	-
Sour	20	48	-
sweet	28	85	-
spicy	106 (42.2)	290 (33.7)	0.883
salty	14	32	-
Skip breakfast (*n* %)			< 0.001^***^
Yes	133 (53.0)	316 (36.7)	-
No	118 (47.0)	545 (63.3)	-
Number of main meals (times/day) (Mean ± SD)	2.5 ± 0.1	2.5 ± 0.0	0.554
Frequency of fast-food (times/week) (Mean ± SD)	2.1 ± 0.1	1.9 ± 0.0	0.047^*^
Frequency of late-night snacks (times/month) (Mean ± SD)	3.0 ± 0.1	2.9 ± 0.0	0.083
Frequency of carbonated soft drink (times/day) (Mean ± SD)	1.5 ± 0.0	1.3 ± 0.0	< 0.01^**^
**Physical activity and sleep habits**			
Sleep duration (h/day) (Mean ± SD)	7.1 ± 0.1	7.2 ± 0.0	0.209
Frequency of physical activity(day/week) (Mean ± SD)	2.9 ± 0.1	2.9 ± 0.0	0.822
Difficulty in falling asleep at night (*n* %)			< 0.001^***^
Yes	89 (35.5)	164 (19.0)	-
No	162 (64.5)	697 (81.0)	-

EAT-26: The Eating Attitudes Test-26. ^*^p < 0.05, ^**^p < 0.01, ^***^p < 0.0001. Mann–Whitney U-test and chi-square test analyses.

### Pearson and Spearman correlation analyses

There was a significant positive correlation between the total scores of MPAI and the total scores of EAT-26 (*r* = 0.245, *p* < 0.001) scales, the frequency of eating fast-food (*r* = 0.180, *p* < 0.001)/late-night snacks (*r* = 0.129, *p* < 0.001), and the frequency of drinking carbonated soft drink (*r* = 0.160, *p* < 0.001), depression and anxiety (*r* = 0.179, *p* < 0.001), difficulty in falling asleep at night (*r* = 0.222, *p* < 0.001), time to stay on the Internet (*r* = 0.273, *p* < 0.001), and time to use a smartphone on weekends (*r* = 0.333, *p* < 0.001)/weekdays (*r* = 0.280, *p* < 0.001). The total scores of the MPAI scale were negatively correlated with skipping breakfast (*r* = −0.211, *p* < 0.001), sleep duration (*r* = −0.089, *p* < 0.01), and the frequency of physical activity (*r* = −0.099, *p* < 0.01) (Table 4).

**Table 4:** Correlations analyses between smartphone addiction and eating disorders and lifestyle.

	**Total scores of MPAI**		**Total scores of EAT-20**		**Difficulty in falling asleep**	
	**r**	***P***	**r**	***P***	**r**	***P***
BMI	−0.006	0.840	−0.024	0.427	−0.019	0.533
Frequency of fast-food	0.180	< 0.001^***^	0.087	< 0.01^**^	−0.55	0.067
Frequency of late-night snacks	0.129	< 0.001^***^	0.100	< 0.001^***^	−0.102	< 0.01^**^
Frequency of carbonated soft drink	0.160	< 0.001^***^	0.033	0.269	0.070	< 0.05^*^
Time to use smartphone weekday	0.280	< 0.001^***^	0.095	< 0.01^**^	−0.113	0.000^***^
Time to use smartphone weekend	0.333	< 0.001^***^	0.088	< 0.05^*^	−0.086	< 0.01^**^
Skip breakfast	−0.0211	< 0.001^***^	−0.016	0.602	0.165	< 0.001^***^
Depression and anxiety	0.179	< 0.001^***^	−0.091	< 0.01^**^	0.188	< 0.001^***^
Sleep duration	−0.089	< 0.01^**^	−0.027	0.369	0.127	< 0.001^***^
Frequency of physical activity	−0.099	< 0.01^**^	0.078	< 0.01^**^	−0.010	0.729
Time to stay on the internet	0.273	< 0.001^***^	0.019	0.517	−0.095	< 0.01^**^
Difficulty in falling asleep at night	0.222	< 0.001^***^	−0.132	< 0.001^***^	-	-
Total score of EAT-20	0.189	< 0.001^***^	-	-	-	-
Total score of MPAI	-	-	-	-	-	-

MPAI, Mobile Phone Addiction Index; EAT-26, The Eating Attitudes Test-26. ^*^p < 0.05, ^**^p < 0.01, ^***^p < 0.001, Pearson and Spearman correlation analyses.

The total scores of EAT-26 were positively associated with the frequency of eating fast food (*r* = 0.087, *p* < 0.01)/late-night snacks (*r* = 0.105, *p* < 0.001), time to use a smartphone on weekends (*r* = 0.088, *p* < 0.01)/weekdays (*r* = 0.095, *p* < 0.01), and the frequency of physical activity (*r* = 0.078, *p* < 0.01). There was a negative correlation between the total scores of the EAT-26 scale and depression and anxiety (*r* = −0.091, *p* < 0.01) and difficulty in falling asleep at night (*r* = −0.132, *p* < 0.001) (Table 4).

In addition, it was observed that difficulty in falling asleep at night was negatively correlated with the frequency of eating late-night snacks (*r* = −0.102, *p* < 0.01), time to use a smartphone on weekends (*r* = −0.086, *p* < 0.01), and time to use smartphones on weekdays (*r* = −0.113, *p* < 0.001). There was a positive relation between the difficulty in falling asleep at night and depression and anxiety (*r* = 0.188, *p* < 0.001), skipping breakfast (*r* = −0.165, *p* < 0.001), and the frequency of drinking carbonated soft drinks (*r* = 0.070, *p* < 0.05) (Table 4).

### Regression analyses

Table 5 shows the multiple linear regression analysis for smartphone addiction. The results indicated that the EAT-26 scores, depression and anxiety, difficulty in falling asleep at night, the frequency of eating fast food/drinking carbonated soft drinks, and time to stay on the Internet, and time to use a smartphone on weekends were the positive predictors of the MPAI scores (*p* < 0.01). The standardized beta coefficients are 0.141 (*p* < 0.001), 0.090 (*p* < 0.01), 0.123(*p* < 0.001), 0.073 (*p* < 0.01), 0.081 (*p* < 0.01), 0.184 (*p* < 0.001), and 0.193 (*p* < 0.001), respectively. In addition, the MPAI scores were negatively associated with skipping breakfast and the frequency of physical activity. The standardized beta coefficients are −0.123 (*p* < 0.001) and −0.061 (*p* < 0.05), respectively.

**Table 5:** Regression analyses for MPAI scores.

**Optimal model**	**R**	**R^2^**	**Adjusted R^2^**	**F**	***P***
	0.520	0.271	0.261	27.145	< 0.001^***^
**Independent variables**			Standardized β	t	*P*
EAT-26 Scores			0.141	5.333	< 0.001^***^
BMI			0.028	1.051	0.293
Depression and anxiety			0.090	3.386	< 0.01^**^
Skip Breakfast			−0.123	−4.150	< 0.001^***^
Frequency of fast-food			0.073	2.698	< 0.01^**^
Frequency of late-night snacks			0.017	0.614	0.539
Frequency of carbonated soft drink			0.081	3.033	< 0.01^**^
Frequency of physical activity			−0.061	−2.266	< 0.05^**^
Difficulty in falling asleep at night			0.123	4.497	< 0.001^***^
Time to stay on the internet			0.184	6.782	< 0.001^***^
Time to use smartphone weekday			0.050	1.456	0.146
Time to use smartphone weekend			0.193	5.591	< 0.001^***^

MPAI, Mobile Phone Addiction Index; EAT-26, The Eating Attitudes Test-26. ^*^p < 0.05, ^**^p < 0.01, ^***^p < 0.001. A multiple linear regression analysis was used.

## Discussion

In this study, we reported the prevalence of smartphone addiction and potential eating disorders. We examined the relationship between smartphone disorder and potential eating disorders and the association between the above two factors and lifestyle, in a large sample of college students in Chengdu, China.

Given the convenience and efficiency that they provide to our daily lives, the number of smartphone users is increasing rapidly. The problem of excessive use of smartphones has also aroused widespread concern in society (51). This survey demonstrated that the prevalence of smartphone addiction tendency among Chinese university students was 22.6%, which was higher than that in the previous studies (52, 53). Our findings suggested that the students with smartphone addiction were more likely to have depression and anxiety and have difficulty in falling sleeping at night. This is consistent with the findings of multiple previous studies, which investigated the relationship between anxiety, depression, sleep disorders, and smartphone addiction. A survey of 319 Turkish university students found positive correlations between the Smartphone Addiction Scale scores and depression levels, anxiety levels, and sleep quality scores (27). A sample of 11,831 Chinese adolescent students demonstrated that time spent on smartphones was associated with an increased risk of clinical depressive symptoms, mediated by sleep disorders (54). Depression was a significant independent positive predictor of smartphone addiction among a sample of 353 Korean college students (25). However, the impact of smartphones on sleep quality is currently rather inconclusive or inconsistent. One study with 844 Belgian adults revealed that smartphone was commonly used as a sleep aid (55), but other studies reported that smartphone use could affect sleep quality (8, 56, 57).

These may be because college students usually have less self-regulatory ability, have relatively weak psychological ability to withstand setbacks and stress resistance, and are more likely to feel lonely, anxious, and depressed (58, 59). According to the results shown in previous studies, most students are aware of their overdependence on smartphones; however, it is difficult to control this behavior by themselves. In addition, it has been pointed out that students with higher depression and anxiety are more likely to use smartphones as a compensatory attachment target (37). Moreover, several studies found that using a smartphone during sleeping hours alters the circadian system, alters cerebral blood flow, and even changes in cardiac rhythms, leading to negative sleep consequences (60). Moreover, current recommendations suggest that smartphones should be at least 3 ft away from the body during sleep (61). In addition, research showed that bedtime procrastination played an intermediary role between smartphone addiction and poor sleep quality (8, 57).

According to the EAT-26 scale, this study indicated that 10.4% of students are at a risk of eating disorders among Chinese college students, which was lower than that reported in previous surveys in other countries (47, 62). Based on Pearson and Spearman correlation analyses and a multiple linear regression analysis, smartphone addiction was positively correlated with eating disorders. In addition, the present study showed that the MPAI scores was significantly positively correlated with the frequency of eating fast food, eating late-night snacks, and drinking carbonated soft drinks and significantly negatively correlated with the frequency of physical activity and sleep duration. Previous studies also suggested that IA and smartphone addiction can lead to changes in lifestyle-related factors, leading to irregular eating habits (26, 35, 47, 63). Literature found that those who are addicted to Internet gaming usually further restricted their eating habits to accommodate their playing. They often miss meals and eat junk food and drink soft drinks while playing online games. Our findings resonate well with the previous studies, which indicated that participants with IA are more likely to consume snacks and carbonated drinks (28, 35). Moreover, this study showed that there is a significant positive correlation between sleep difficulty and skipping breakfast in college students. Hence, poor sleep quality may play an intermediary role between smartphone addiction and kipping breakfast.

## Limitation

There are several limitations of this study. First, the descriptive, cross-sectional design did not enable us to establish a causal relationship between MPAI scores and eating disorders or lifestyle. Second, the study was conducted only among college students. In addition, many variables such as smartphone use, emotional states, eating habits, and lifestyles were self-reported and, therefore, may have some response bias. Despite those limitations, the major strength of our study was the simultaneous evaluation of the relationship between smartphone addiction and eating disorders as well as lifestyle. Furthermore, we compared the proportion of smartphone addiction by self-reporting and assessment using the MPAI scales. The findings of this study provided important data on identifying the association between smartphone addiction and eating disorders as well as lifestyle. This information can be used as a reference for public health decision-makers and healthcare practitioners to develop appropriate strategies and intervention plans to control smartphone addiction among adolescent college students. In the future, more longitudinal studies on smartphone addiction are needed.

## Conclusion

Our findings confirmed the close associations between smartphone addiction and eating disorders as well as between eating habits and lifestyles among college students. In accordance with the results, early screening and management of smartphone use among university students are recommended. Parents and teachers should pay more attention to their mental state to cultivate healthy eating habits and lifestyle characteristics and to improve their abilities of self-regulation to help in lowering the risk of smartphone addiction. The modification of dietary habits and lifestyle factors needs to be considered when developing strategies and interventions to prevent smartphone addiction among college students.

## Data availability statement

The original contributions presented in the study are included in the article/Supplementary material, further inquiries can be directed to the corresponding authors.

## Ethics statement

The studies involving human participants were reviewed and approved by the Sichuan Traditional Chinese Medicine Regional Ethics Review Committee (No.2021KL-044). The patients/participants provided their written informed consent to participate in this study.

## Author contributions

JW, T-MZ, and LZ conceived and designed the study. JW and Q-HH drafted the manuscript. JW, WP, and YT participated in the recruitment and data collection. All authors read and approved the final manuscript.
