# Supplementary material for: Relationship between smartphone addiction and eating disorders and lifestyle among Chinese college students
Source: Front Public Health. 2023 May 19;11:1111477. doi: 10.3389/fpubh.2023.1111477 (PMC10235600; doi:10.3389/fpubh.2023.1111477)
Supplement: Supplementary file 1 [file Table_1.DOCX]

The details of Lifestyle variables

| **Eating habits** |
| --- |
| 1.Do you have a taste preference? |
| No |
| Yes |
| Like the sour taste |
| Like the sweet |
| Like spicy |
| Like salty |
| 2. How many times do you eat main meals every day? (time/day) |
| 1 |
| 2 |
| 3 |
| 4 |
| 3. Do you eat breakfast regularly? |
| Yes |
| No |
| 4. How many times a week do you eat fast food? (time/week) (hamburgers, instant noodles, snail noodles, hot and sour noodles, etc.) |
| 1 |
| 2 |
| 3 |
| >=4 |
| 5. In the last month, how many times a week have you had late-night snacks? (time/week) |
| <3 |
| 3-4 |
| >=5 |
| 6. In the last month, how many times a day have you consumed carbonated beverages such as Coca-Cola, Sprite or Pepsi?" (excluding diet soft drinks) (time/month) |
| <1 |
| >=1 |
| **Physical activity and sleep habits** |
| 7. How many days during the week did you exercise for a total of at least 60 minutes per day? (day/week) |
| <3 |
| 3-4 |
| >=5 |
| 8. How many hours do you sleep on average every day? (h/day) |
| <6 |
| 6-7 |
| 8-9 |
| >9 |
| 9. Do you often have trouble falling asleep? |
| Yes |
| No |
